# HIV Productively Infects Highly Differentiated and Exhausted CD4+ T Cells During AIDS

**DOI:** 10.20411/pai.v8i2.638

**Published:** 2024-02-22

**Authors:** Clayton Faua, Axel Ursenbach, Anne Fuchs, Stéphanie Caspar, Frédérick Jegou, Yvon Ruch, Baptiste Hoellinger, Elodie Laugel, Aurélie Velay, David Rey, Samira Fafi-Kremer, Pierre Gantner

**Affiliations:** 1 INSERM UMR_S1109, Strasbourg University, Strasbourg, France; 2 Le Trait d'Union, HIV-Infection Care Center, Strasbourg University Hospital, Strasbourg, France; 3 Clinical Virology Laboratory, Strasbourg University Hospital, Strasbourg, France; 4 Infectious Diseases Department, Strasbourg University Hospital, Strasbourg, France

**Keywords:** AIDS, recent infection, productively infected cells, phenotype, exhaustion

## Abstract

**Background:**

Throughout HIV infection, productively infected cells generate billions of viral particles and are thus responsible for body-wide HIV dissemination, but their phenotype during AIDS is unknown. As AIDS is associated with immunological changes, analyzing the phenotype of productively infected cells can help understand HIV production during this terminal stage.

**Methods:**

Blood samples from 15 untreated viremic participants (recent infection, n=5; long-term infection, n=5; active opportunistic AIDS-defining disease, n=5) and 5 participants virologically controlled on antiretroviral therapy (ART) enrolled in the Analysis of the Persistence, Reservoir and HIV Latency (APRIL) study (NCT05752318) were analyzed. Cells expressing the capsid protein p24 (p24+ cells) after 18 hours of resting or 24 hours of stimulation (HIV-Flow) revealed productively infected cells from viremic participants or translation-competent reservoir cells from treated participants, respectively.

**Results:**

The frequency of productively infected cells tended to be higher during AIDS in comparison with recent and long-term infections (median, 340, 72, and 32/million CD4+ T cells, respectively) and correlated with the plasma viral load at all stages of infection. Altogether, these cells were more frequently CD4^low^, HLA-ABC^low^, CD45RA-, Ki67+, PD-1+, with a non-negligible contribution from pTfh (CXCR5+PD-1+) cells, and were not significantly enriched in HIV coreceptors CCR5 nor CXCR4 expression. The comparison markers expression between stages showed that productively infected cells during AIDS were enriched in memory and exhausted cells. In contrast, the frequencies of infected pTfh were lower during AIDS compared to non-AIDS stages. A UMAP analysis revealed that total CD4+ T cells were grouped in 7 clusters and that productive p24+ cells were skewed to given clusters throughout the course of infection. Overall, the preferential targets of HIV during the latest stages seemed to be more frequently highly differentiated (memory, T_TD_-like) and exhausted cells and less frequently pTfh-like cells. In contrast, translation-competent reservoir cells were less frequent (5/million CD4+ T cells) and expressed more frequently HLA-ABC and less frequently PD-1.

**Conclusions:**

In long-term infection and AIDS, productively infected cells were differentiated and exhausted. This could indicate that cells with these given features are responsible for HIV production and dissemination in an immune dysfunction environment occurring during the last stages of infection.

## INTRODUCTION

During the last decades, HIV-infected CD4+ T cells have been extensively studied, unraveling their heterogeneity in terms of phenotype [1–6], proviral sequence [7-12], and capacity to produce infectious viral particles [6, 13–16]. HIV establishes 2 forms of infection: either CD4+ T cells are infected productively and thus produce infectious virions [17], or cells are infected latently and thus produce none or few HIV transcripts and proteins [15]. While latently infected cells survive for decades *in vivo* [13] and are responsible for the viral rebound when antiretroviral therapy (ART) is interrupted [18], productively infected cells have a half-life of approximately 2 days but produce billions of viral particles and are responsible for virus dissemination body-wide [19, 20]. We recently showed that the phenotype of productively infected cells is constantly modified throughout the course of acute infection and also differs in chronically infected individuals [21]. However, the late cellular targets of HIV after multiple virological failures and during the final stage of HIV infection (AIDS) remain uncharacterized.

First, we know that secondary lymphoid organs' structure and T-cell phenotype are modified with disease progression and thus differ during AIDS from what we can observe in acute infection. During AIDS, the architecture of lymph nodes is disrupted: germinal centers and follicular dendritic cell networks disappear [22–24]. Similarly, T follicular helper (Tfh) cells were depleted in an AIDS/SIV model [25]. In humans, CXCR5 expression on blood CD4+ T cells decreases over time in HIV progressors [26], and peripheral (or circulating) Tfh (pTfh)[27] cell population frequencies decrease during disease progression [28]. As Tfh cells represent essential contributors to the pool of HIV-infected cells during chronic infection [21, 29], changes affecting the Tfh and pTfh populations during AIDS could affect the ways HIV virions are produced as well. Indeed, it has been hypothesized that HIV replication mostly arises from lymph nodes during chronic infection and, conversely, from blood during AIDS [24].

Second, immune exhaustion and activation during HIV infection have been well documented [30]. As an example, expression of immune checkpoint molecules PD-1 and TIGIT on CD4+ T cells was shown to increase with time during untreated infection [31, 32] and was inversely correlated with the CD4+ T-cell count during AIDS [33]. CD4+ T cells from people with AIDS show the highest level of activation when compared to non-AIDS participants [34]. Therefore, the frequency of naïve cells decreases during AIDS [35]. As productively infected cells tend to be enriched in memory, activated, and exhausted cells [6, 21], changes occurring during AIDS might also impact HIV production.

Finally, it is also documented that HIV variants evolve to a tropism switch from CCR5-tropic strains to CXCR4-tropic strains with disease progression [36]. Although CXCR4 expression on CD4+ T cells does not vary during AIDS [37], CCR5 expression is increased compared to HIV-infected non-AIDS individuals [37]. The expression of CCR5 and CXCR4 on CD4+ T cells was shown to be reciprocal: naïve cells express CXCR4 while effector memory cells express CCR5 [38]. Although it was recently shown that the tropism switch did not originate from target cell depletion [39], the tropism switch could have an impact on preferential infection and, thus, the phenotype of productively infected cells.

Altogether, the impact of AIDS phenotypic changes in CD4+ T cells on the pool of productively infected cells and their contribution to the late dissemination of HIV during advanced disease are currently unknown. Here, we used a phenotypic analysis on productively HIV-infected cells in blood from individuals at advanced stages of HIV infection to investigate the location of productive infection during AIDS.

## METHODS

### Participants

Peripheral blood mononuclear cells (PBMCs) samples collected from HIV-infected viremic participants at different stages of HIV infection were analyzed (n=20). Among the 15 untreated viremic individuals, participants were stratified as follows: recent infection (n=5; acute or early, < 6 months of infection), long-term infection (n=5; virological failure or late diagnosis, > 7 years of infection, based on serological tests and/or participants' declaration), and AIDS stage (n=5, defined as an ongoing opportunistic AIDS-defining disease as per the CDC staging). In the case of virological failure, treatment interruption occurred upon participants' own decisions. Also, the seropositivity duration of some ‘AIDS’ participants was unknown due to late diagnosis. Additional PBMCs were collected from HIV-infected individuals undergoing successful (HIV-1 RNA <50 copies/mL) ART (n=5) and from HIV-uninfected individuals (HIV-, n=5). Clinical and therapeutic data associated with these samples were also collected (Table 1). Virological data included tropism determination obtained using the sequencing protocol and algorithm recommended by the ANRS (*Agence Nationale de Recherche sur le SIDA*) rules (10/2022, https://hivfrenchresistance.org/).

**Table 1.** HIV-Infected Participants' Characteristics

Group	#ID	Age (years)	Sex	Sub-type	Tropism	Known seropositivity (months)	Viral load (log_10_ copies/mL)	Indetect-ability duration (months)	CD4 count (/mm^3^)	CD4/CD8 ratio	Opportunistic disease(s)	ART treatment
Recent	1	58	F	A6	R5	0.4 (Fiebig VI)	4.84	/	223	0.42	/	/
	2	27	M	B	R5	3 (Fiebig VI)	5.27	/	932	0.66	/	/
	3	58	M	CRF02	R5	0.25 (Fiebig II)	>7.00	/	223	0.64	/	/
	4	37	M	CRF02	R5	1.5 (Fiebig V)	1.89	/	387	0.49	/	/
	5	24	M	CRF02	R5	0.5 (Fiebig IV)	6.32	/	365	0.25	/	/
Long	6	57	M	CRF01	X4	361	4.71	/	47	0.11	/	/
	7	41	F	A1	R5	201	2.96	/	313	0.33	/	/
	8	38	F	D	R5X4	237	4.44	/	411	0.81	/	/
	9	41	M	A6	R5	0.25	3.44	/	141	0.14	/	/
	10	56	M	F2	R5	89	4.76	/	518	0.69	/	/
AIDS	11	46	M	B	R5	0.4	5.50	/	56	0.10	CMV retinitis	/
	12	45	M	A6	X4	0	5.91	/	44	0.12	-Pneumocystis -Cryptococcosis	/
	13	57	M	CRF02	R5	0.4	6.00	/	43	0.07	-Esophageal candidiasis -Pneumocystis	/
	14	44	M	CRF18	R5	102	6.18	/	20	0.04	HIV encephalitis	/
	15	61	F	CRF18	X4	319	4.98	/	22	0.02	-Esophageal candidiasis -Pneumocystis	/
ART	16	50	M	B	/	240	<1.48	231	553	0.59	/	RPV/DTG
	17	36	M	A	/	49	<1.48	48	255	0.57	/	3TC/DTG
	18	39	F	CRF18	/	78	<1.48	69	489	0.82	/	BIC/TAF/FTC
	19	41	F	A	/	181	<1.48	174	718	0.77	/	RPV/TDF/FTC
	20	55	M	CRF02	/	52	<1.48	6	962	1.69	/	DRV/r+TDF FTC +DOR

ART, antiretroviral therapy; M, male; F, female; RPV, rilpivirine; DTG, dolutegravir; 3TC, lamivudine; BIC, bictegravir; TAF, tenofovir alafenamide; FTC, emtricitabine; TDF, tenofovir disoproxil fumarate; DRV/r, darunavir/ritonavir; DOR, doravirine.

### Ethics

Participants were included in the APRIL study (Analysis of the Persistence, Reservoir and HIV Latency). This monocentric, observational, and prospective study was approved by the institutional review board CPP (*Comité de Protection des Personnes*) Est I on January 5, 2023 (2022-A02567-36) and promoted by the University Hospitals of Strasbourg, France (NCT05752318). This study is aimed at describing HIV-infected cells from individuals followed at our institution (*Le Trait d'Union*, HIV-infection care center, and Infectious Diseases Department). All participants provided informed consent for their data and samples to be used for biological research. The study consists of a single visit during which 48 mL of peripheral blood is drawn. Approximately 50 to 100x10^6^ PBMCs are isolated and frozen within 4 hours of collection.

### Cell Culture

The HIV-Flow assay (Supplemental Figure 1A) was used to quantify and analyze the phenotype of cells expressing the viral p24 capsid protein [6, 21]. Briefly, CD4+ T cells were isolated by negative magnetic selection using the EasySep Human CD4+ T Cell Enrichment Kit (StemCell Technology, Cat#19052). Purity was typically >98%. 5-15x10^6^ CD4+ T cells were resuspended at 2x10^6^ cells/mL in RPMI + 10% Fetal Bovine Serum, and antiretroviral drugs were added to the culture medium (200nM raltegravir, 200nM lamivudine). Cells were then rested (18 hours at 37°C 5% CO_2_) for samples from viremic participants, allowing us to reveal productively infected cells.

Since the HIV-infected cells remaining after a successful ART treatment are latently infected reservoir cells, stimulation was required to force the production of the capsid p24. To this end, CD4+ T cells from ART-treated participants were treated with 5µg/mL Brefeldin A (BFA, Sigma, Cat#7651) for 1 hour and were then stimulated for 24 hours (37°C 5% CO_2_) with 162nM Phorbol-12-myri-state-13-acetate (PMA, Sigma, Cat#P8139) and 1µg/mL ionomycin (Sigma, Cat#I9657).

### Flow Cytometry

Cells were collected, resuspended in PBS, and stained with the Aqua Live/Dead staining kit for 20 minutes at 4°C. Cells were then stained with antibodies against extracellular molecules in PBS + 4% human serum (Sigma, Cat#H4522-100ML) for 20 minutes at 4°C. After a 45-minute fixation/permeabilization step performed with the FoxP3 Transcription Factor Staining Buffer Set (eBioscience, Cat#00-5523-00) following the manufacturer's instructions, cells were stained with anti-p24 KC57 and anti-p24 28B7 antibodies for an additional 45 minutes at room temperature in the FoxP3 Buffer. Cells were then washed and resuspended in PBS for subsequent analysis.

Dual recognition by both p24 antibodies defined infected p24+ cells (Supplemental Figure 1B) and allowed for analysis of their phenotype (Supplemental Figure 1C). In samples from viremic participants, p24+ cells revealed after the 18 hours of resting were defined as productively infected cells. In samples from ART-treated participants, p24+ cells revealed after the 24-hour stimulation were defined as latently infected cells (translation-competent HIV reservoir). An example of p24 expression dot plots is shown for each group of participants (HIV-, recent infection, long-term infection, AIDS, and ART) in Supplemental Figure 1D.

p24 KC57-PE was purchased from Beckman Coulter (Cat#6604667, Dilution 1/1000) and p24 28B7-APC from MediMabs (Cat#MM-0289-APC, Dilution 1/1000). CD4-APC-H7 (Clone: RPA-T4, Cat#560168, Dilution 1/100), CD45RA-BV605 (Clone: HI100, Cat#562886, Dilution 1/50), Ki67-PerCP-Cy5.5 (Clone: B56, Cat#561284, Dilution 1/25), and HLA-ABC-BV421 (Clone: W6/32, Cat#567861, Dilution 1/50) were purchased from BD Bioscience. CXCR5-FITC (Clone: J252D4, Cat#356914, Dilution 1/50), CXCR4-PE/Dazzle594 (Clone: 12G5, Cat#306526, Dilution 1/33), CCR5-BV711 (Clone: J418F1, Cat#359130, Dilution 1/25), TIGIT-PE-Cy7 (Clone: A15153G, Cat#372714, Dilution 1/50), and PD-1-A700 (Clone: EH12.2H7, Cat#329952, Dilution 1/25) were purchased from BioLegend. Live/Dead Aqua Cell Stain (405nm) was purchased from ThermoFisher Scientific (Cat#L34957).

Flow cytometry data of p24+ cells were analyzed using FlowJo version 10.9.0. To gather an overview of the immune populations present in the pool of CD4+ T cells in participants in recent infections, long-term infections, and AIDS, 20,000 p24-cells were downsampled for each participant, and the totality of p24+ cells were added. Flow cytometry data were visualized using uniform manifold approximation and projection (UMAP)-dimensionality reduction on the total concatenated pool of p24-/p24+ cells from each participant [40]. Combined p24- and p24+ cells were then further grouped using FlowSOM, a self-organizing map that clusters cells depending on their overall similarities in marker expression [41].

Of note, PMA/ionomycin stimulation was shown to downregulate the expression of some surface markers [6]. To determine which markers are affected, the phenotype of CD4+ T cells from healthy donors (n=5) was analyzed *ex vivo* or with 24-hour BFA + PMA/ionomycin stimulation (Supplemental Figure 2A). Although this short stimulation did not affect cell viability, the expression of CD4, CCR5, CXCR4, TIGIT, and CXCR5 was down-modulated. Therefore, only the expression of HLA-ABC, CD45RA, Ki67, and PD-1 was analyzed in ART participants.

### Data Representations and Statistical Analyses

Data were analyzed and represented using GraphPad Prism version 9.5.1. As indicated in the figure legends, individual results were represented with median and range. Correlations were determined using non-parametric Spearman's test. For comparisons, non-parametric Wilcoxon matched-pairs signed rank, or Mann-Whitney tests were used. *P* values of less than or equal to 0.05 were considered statistically significant.

## RESULTS

### Participant Characteristics

Participants (n=20) were mostly men (80%) with a median age of 45 years (range 24 to 61) and were infected by various HIV subtypes (Table 1). The 5 participants with recent infection were all infected with CCR5-tropic strains and displayed Fiebig stages from II to VI. In comparison, the long-term infected and AIDS participants displayed both CCR5 and CXCR4-tropic strains. However, due to late diagnosis, the exact infection duration is unknown but was estimated based on case history to be over 7 years for both long-term infection and AIDS groups. The median viral loads were 5.27, 4.44, and 5.91 log_10_ copies/mL during recent, long-term infections, and AIDS, respectively, and undetectable in participants under ART. Viral loads were significantly higher during AIDS when compared to long-term infection (Mann-Whitney, *P*=0.008). The median CD4+ T-cell counts were 365, 313, 43, and 553/mm^3^ during recent, long-term infections, AIDS, and under ART, respectively. The CD4 count was significantly lower during AIDS compared to both recent and long-term infections (*P* =0.01).

### The Frequencies of Productively Infected Cells Correlate with Plasma Viral Load

**Figure 1.**
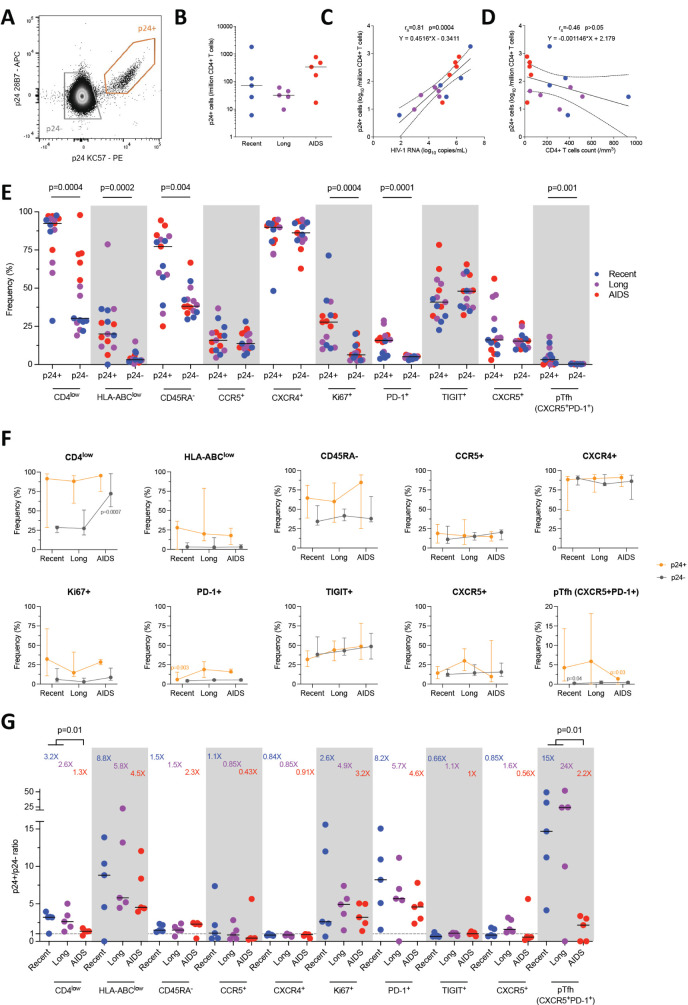
**The phenotype of productively infected p24+ cells differs from p24-cells and changes over time**. A) Representative dot plot showing the gating strategy for identification of productively infected p24+ cells (orange gate) and p24-cells (grey gate). B) Frequency of p24+ cells per group (median). C-D) Correlations between p24+ cells frequencies and plasma viremia (C) or CD4+ T-cell count (D). The regression line was plotted with 95% CI, and correlation was assessed (Spearman). E) Frequencies of p24+ and p24-cells expressing each marker or combination of markers (CD4^low^, HLA-ABC^low^, CD45RA-, CCR5+, CXCR4+, Ki67+, PD-1+, TIGIT+, CXCR5+, peripheral T follicular helpers [pTfh] cells [CXCR5+PD-1+]) are depicted for each participant (n=15) with median. Significant changes are highlighted with the *P* value (Wilcoxon). F) The frequency of p24+ (orange lines) and p24-(grey lines) cells expressing each marker or combination of markers is depicted for each stage of infection. Median values are plotted with range. Significant changes are highlighted with the *P* value of the differing point (Mann-Whitney). G) Ratio of frequencies of cells (p24+/p24-) expressing each marker or combination of markers according to infection stage are depicted for each participant (n=15) with a median. Significant changes are highlighted (Mann-Whitney).

The frequency of HIV-infected cells has been detected using the HIV-Flow assay (Figure 1A) [6]. Median p24+ cell frequencies were 72, 32, and 340/million CD4+ T cells during recent, long-term infections, and AIDS, respectively (Figure 1B). The frequencies of p24+ cells tended to be higher during AIDS when compared to long-term infection (Mann-Whitney, *P*=0.09) but were similar to frequencies observed during recent infection (*P*=0.42). To evaluate if these changes were linked to HIV stages, we compared p24+ cell frequencies with plasma viral load (Figure 1C) and CD4+ T-cell count (Figure 1D). A significant positive correlation between the frequencies of p24+ cells and the viral load (Spearman, r_s_=0.81, *P*=0.0004) and a trend toward a negative correlation between p24+ frequencies and the CD4 count (r_s_=-0.46, *P*=0.08) were observed. Of note, frequencies of p24+ cells were highly variable during recent infection. These variations may be due to highly different viral loads and CD4 counts across Fiebig stages. As an example, the highest p24+ cell frequency was observed in the participant in Fiebig II stage, which corresponds to peak viremia associated with a sharp decrease in the CD4 count.

### Productively Infected p24+ Cells Display a Skewed Phenotype

The phenotype of HIV-infected cells was analyzed by comparing markers expression in p24+ cells and non-producing (p24-) cells (Figure 1E). As expected, the productively infected p24+ cells expressed lower levels of CD4 (Wilcoxon, *P*=0.0004) and HLA-ABC (*P*=0.0002) than p24-cells. They also displayed more frequently a memory phenotype (CD45RA-, *P*=0.004) and a proliferative state (Ki67+, *P*=0.0004) compared to p24-cells. Interestingly, the expression of HIV coreceptors CCR5 and CXCR4 was not significantly different between p24+ and p24-cells. The expression of the exhaustion marker PD-1 (but not TIGIT) was significantly higher in p24+ cells compared to p24-(*P*=0.0004). The expression of CXCR5 alone was similar between p24+ and p24-cells. Albeit at low frequencies, pTfh cells (CXCR5+PD-1+) represented a non-negligible part of HIV-infected cells (*P*=0.001). Altogether, p24+ cells were frequently CD4^low^, HLA-ABC^low^, CD45RA-, Ki67+, some of which also were PD-1+ and pTfh cells.

As participants were infected with various HIV-1 subtypes, we compared the phenotype of p24+ and total CD4+ T cells according to HIV clade (Supplemental Figure 3A-B). Although it is impossible to perform statistical comparisons, there were no trends toward phenotype variability between clades. On the other hand, as 4 participants were infected with R5X4 dual tropic (n=1) and X4 tropic variants (n=3), we also compared the phenotype of p24+ and total CD4+ T cells according to HIV tropism (Supplemental Figure 3C-D). Overall, the phenotypes were similar, but p24+ cells from R5X4/X4-infected participants expressed more frequently TIGIT, and CD4+ T cells expressed more frequently CXCR4.

### The Phenotype of Productively Infected Cells Changes Between HIV Stages

We then compared the expression of each marker in productively infected p24+ cells and total CD4+ T cells with the viral load (Supplemental Figure 3) and the CD4 count (Supplemental Figure 4) during the 3 HIV stages. Firstly, the frequency of CD4^low^ in p24+ cells positively correlated with the viral load (Spearman, r_s_=0.73, *P*=0.003) but not in CD4+ T cells. Inversely, the frequency of CD45RA-displayed a negative correlation in CD4+ T cells (r_s_=-0.55, *P*=0.03) but not in p24+ cells. The frequency of Ki67+ in CD4+ T cells was positively correlated with the viral load (r_s_=0.62, *P*=0.01) but not in p24+ cells. Secondly, in CD4+ T cells, the frequency of CD4^low^ was inversely correlated with the CD4 count (r_s_=-0.94, *P*<0.0001), while no correlation was seen in p24+ cells. Interestingly, the frequency of HLA-ABC^low^ in CD4+ T cells did not correlate with the CD4 count but correlated positively in p24+ cells (r_s_=0.55, *P*=0.03). The frequency of Ki67+ was also negatively correlated in CD4+ T cells (r_s_=-0.62, *P*=0.01). As expected, the expression of the exhaustion marker TIGIT displayed a negative correlation with the CD4 count in both CD4+ T cells (r_s_=-0.68, *P*=0.007) and p24+ cells (r_s_=-0.58, *P*=0.02). Finally, the pTfh frequency was inversely correlated with CD4 count (r_s_=-0.58, *P*=0.02) in p24+ cells but not in CD4+ T cells.

Given these correlations, we sought to compare the markers expressed by productively infected p24+ and p24-cells between these three stages of infection (Figure 1F). No significant differences were observed for 2-by-2 comparisons. However, the frequency of CD4^low^ p24-cells was significantly higher during AIDS compared to combined recent and long-term infections (Mann-Whitney, *P*=0.0007), probably due to the extreme immune dysregulation observed during the terminal stage of infection. In p24+ cells, PD-1 expression was lower in recent infection compared to combined long-term infection and AIDS (*P*=0.003). As for pTfh cells, their frequency in p24+ cells was significantly higher in combined recent and long-term infections compared to AIDS (*P*=0.03). Overall, frequencies of PD-1 expression and pTfh contribution to the pool of productively infected cells varied significantly between HIV stages, while the frequencies of the expression of other markers remained stable.

To ensure that these minor variations were explicitly attributable to p24+ cells, we then calculated the ratio of each marker's expression between p24+ and p24-cells (Figure 1G). Ratios varied widely according to markers and stages (from 0.43X to 24X). The highest enrichment ratios were observed for pTfh and were significantly higher in combined recent and long-term infections compared to AIDS (15X, 24X, and 2.2X, respectively; Mann-Whitney, *P*=0.01). Moreover, the enrichment ratios of HLA-ABC^low^ (8.8-4.5X) and PD-1+ (8.2-4.6X) were also high but not significant between the stages. The enrichment ratios of CD45RA-(1.5-2.3X) and Ki67+ (2.6-3.2X) were lower and were not significant either between the stages. Expectedly, the enrichment of CD4^low^ was higher in recent and long-term infections when compared to AIDS (3.2X, 2.6X, and 1.3X, respectively, *P*=0.01). Altogether, this shows that enrichment factors vary throughout the infection independently of the frequency of target cells.

### Despite Phenotypic Changes, HIV-1 Preferentially Infects the Same Cell Populations Throughout the Course of Infection

**Figure 2.**
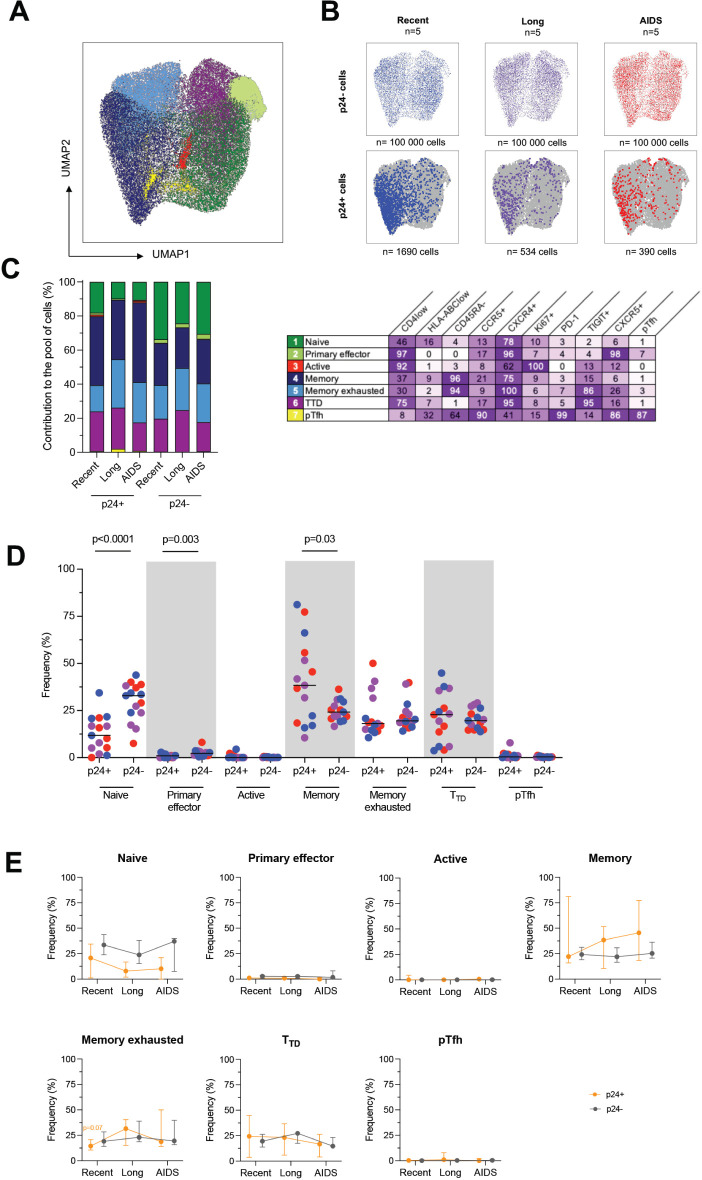
**Productively infected p24+ cells are skewed to given clusters throughout the course of infection**. A) p24+ cells and a fraction of p24-cells phenotypic data were integrated in a UMAP analysis and generated 7 cell clusters. B) Dot plots of the UMAP analysis of all cells (in grey) as shown in panel A, on which the p24- and p24+ cells by stage of infection are overlaid in colors. Numbers of samples analyzed are indicated at the top of the dot plots, and the number of cells is indicated at the bottom. C) The frequency of each of these clusters among p24+ and p24-cells is depicted in bar graphs according to the stage of infection. Numbers of samples analyzed are as in B. The table shows the name of the identified clusters hypothesized according to their phenotype and the detailed marker expression frequencies for each cell cluster (%). D) Frequencies of p24+ and p24-cells included in each cluster are depicted for each participant (n=15) with a median. Significant changes are highlighted with the *P* value (Wilcoxon). E) The frequency of p24+ (orange lines) and p24-(grey lines) cells included in each cluster is depicted for each stage of infection. Median values are plotted with range. Changes close to significance are highlighted with the *P* value of the trending point (Mann-Whitney).

Next, we evaluated the markers that productively infected p24+ cells were co-expressing to assert more specifically their phenotype all along HIV infection. To this end, we realized a UMAP analysis on cells gathered from all the viremic participants. Since p24+ are not frequent, we gathered all of them from all participants (n=2614 p24+ cells). As for p24-cells, we downsampled 20,000 of them from every participant (n=300,000 p24-cells) to have the same amount for each of them. Cells were grouped in 7 clusters by the similarities of their markers' expression (Figure 2A). We then revealed the p24+ and p24-cells distributed in these clusters (Figure 2B) and observed that these cells were well localized in given clusters. The clusters in which p24+ cells were found seemed to change between HIV stages. Thus, we calculated for each stage the frequencies of clusters in which p24+ and p24–cells were distributed and determined the phenotype of each cluster (Figure 2C). However, since the analysis was based on the expression of limited markers, the suspected phenotypes might be incomplete. Overall, the cells that mostly contributed to the total pool of cells were equally naïve (25.1%), memory (29.4%), memory exhausted (20.2%), and terminally differentiated T-like cells (T_TD_, 22.3%). With lower proportions, primary effector-like (2.43%), proliferative (0.18%), and pTfh-like cells (0.4%) also contributed to the total pool of cells. By comparing the frequency of each population between p24+ and p24-cells (Figure 2D), we showed that the frequency of naïve and primary effectors cells was significantly lower in p24+ cells when compared to p24-cells (Wilcoxon, *P*<0.0001 and *P*=0.003, respectively). Conversely, the frequency of memory cells was higher in p24+ cells when compared to p24-cells (*P*=0.03). We also sought to evaluate the variations of each population frequency between HIV stages in both p24+ and p24-cells (Figure 2E). No significant differences were observed for 2-by-2 comparisons. However, we only observed a trend toward a higher frequency of memory exhausted cells in recent infection compared to combined long-term infection and AIDS (*P*=0.07).

### The Frequencies of Translation-Competent Latently Infected Cells Correlate with the CD4 count

**Figure 3.**
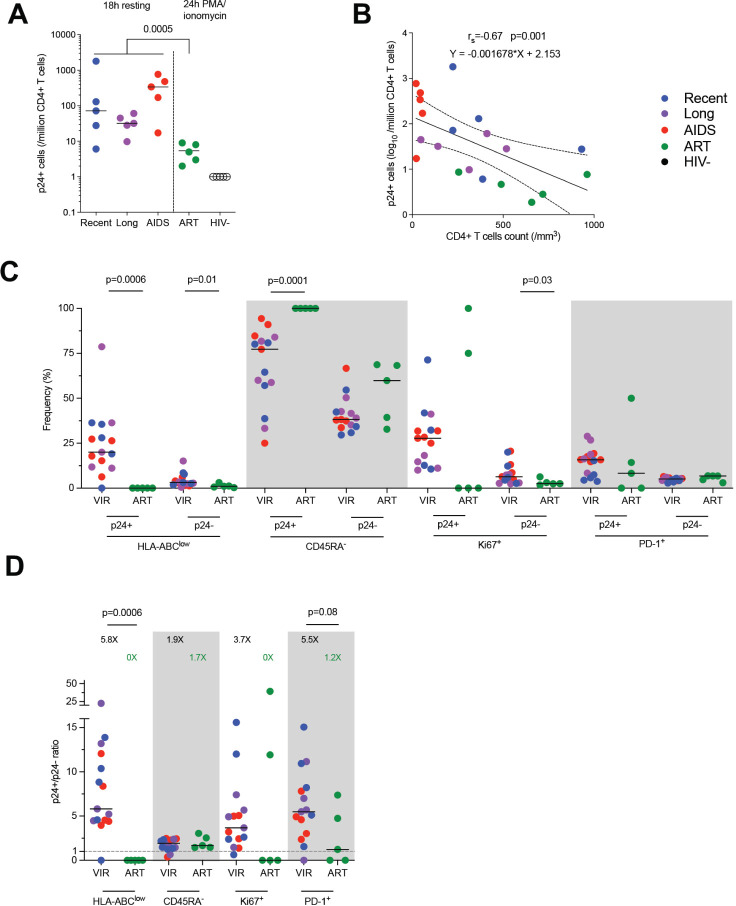
**The phenotype of translation-competent latently infected cells differs from productively infected cells**. A) Frequency of p24+ cells per group (median). Productively infected cells were more frequent than translation-competent latently infected cells (Mann-Whitney). B) Correlation between p24+ cells frequencies and CD4+ T-cell count. The regression line was plotted with 95% CI, and correlation was assessed (Spearman). C) Frequencies of p24+ and p24-cells expressing each marker or combination of markers (HLA-ABC^low^, CD45RA-, Ki67+, PD-1+) are depicted for each participant (n=20) with median. Significant differs between viremic untreated and ART-treated participants are highlighted with the *P* value (Wilcoxon). D) Ratio of frequencies of cells (p24+/p24-) expressing each marker or combination of markers according to infection stage are depicted for each participant (n=20) with a median. Significant or close to significance changes are highlighted with *P* value (Mann-Whitney).

Using BFA + PMA/ionomycin stimulation for 24 hours, we were able to detect translation-competent reservoir cells expressing p24 (p24+ cells) from ART-treated participants (Figure 3A). As expected [6], the median frequency of p24+ cells in ART-treated participants was 5/million CD4+ T cells and was significantly lower when compared to productively infected cells (Mann-Whitney, *P*=0.005). We also combined the frequencies of p24+ cells from all the groups to compare with the CD4+ T-cell count (Figure 3B) and found a negative correlation (Spearman, r_s_=-0.67, *P*=0.001).

### The Phenotype of Latently Infected Cells Differs From That of Productively Infected Cells

We next sought to compare the expression of each of these markers in latently infected p24+ cells and total CD4+ T cells with the CD4 count (Supplemental Figure 6). We observed that, in total CD4+ T cells, the frequency of HLA-ABC^low^ cells was negatively correlated with the CD4 count (Spearman, r_s_=-1, *P*=0.02). We then compared the expression of HLA-ABC, CD45RA, Ki67, and PD-1 in both p24+ and p24-cells between untreated viremic and ART-treated participants (Figure 3C). First, the frequencies of HLA-ABC^low^ were significantly lower under ART in p24+ and p24-cells (Mann-Whitney, *P*=0.0006 and *P*=0.01, respectively).

Also, the expression of CD45RA was completely absent in the latently infected p24+ cells of ART-treated individuals, thus being significantly different from the productively infected cells (*P*=0.0001). Ki67 expression was also lower on ART in p24-cells (*P*=0.03), compatible with a lower state of immune activation during ART. To test whether these changes were specific to latently infected cells, we analyzed the ratio of expression of each marker between p24+ and p24-cells (Figure 3D). We confirm that reservoir cells were more enriched in HLA-ABC expressing cells on ART but saw no difference in CD45RA expression enrichment between treated and untreated participants. Of note, reservoir cells also tend to be less enriched in PD-1 expressing cells when compared to productively infected cells.

## DISCUSSION

During HIV infection, productively infected cells play a critical role in HIV pathogenesis: (1) they are responsible for rapid and sustained HIV production and dissemination throughout the whole body, (2) they are the main targets of HIV-specific CD8+ T cells, and (3) some of them will become reservoir cells due to post-activation latency [42, 43]. Analyzing their phenotype could thus represent an interesting way to understand the mechanisms of these processes and design future cure strategies. Though it has been recently documented in acute and chronic infections, the phenotype of productively infected cells remains undetermined during advanced disease stages such as AIDS. Indeed, the anatomical changes associated with the AIDS stage, particularly in secondary lymphoid organs, might impact their phenotype. To this end, we used HIV-Flow assay to assess the phenotype of p24-producing cells in people living with HIV with an ongoing opportunistic disease (AIDS) and compared them to earlier stages of infection.

When considering all stages together, we noted that p24+ cells were more frequently CD4^low^, HLA-ABC^low^, CD45RA-, Ki67+, PD-1+, with some of them being pTfh cells, compared to their p24-counterparts. On the one hand, in concordance with the literature, the low surface expression of CD4 and HLA-ABC is most likely due to internalization and degradation mainly directed by the viral protein nef [44, 45]. The enrichment of CD4^low^ was significantly lower during AIDS compared to non-AIDS stages, likely due to a higher frequency of CD4^low^ in all cells, while it remained stable in p24+ cells between HIV stages. In contrast, CD4^low^ frequencies in total CD4+ T cells correlated with the CD4 count. More specifically, AIDS individuals displayed the highest frequency of CD4^low^ and the lowest CD4 count, which suggests a higher CD4 downregulation during AIDS. This is in line with previous studies. Indeed, CD4 downregulation has been described as higher during AIDS [46] and was not associated with viral load set points and CD4 decline *in vitro* [47]. Moreover, the frequencies of CD4^low^ in p24+ cells were positively correlated with the plasma viral load. As stated in the literature, cell surface expression of CD4 is associated with an inhibition of the release of infectious virions, as CD4 hypothetically retains budding virions. Therefore, a hypothesis could be that the nef-mediated downregulation of CD4 detected in infected cells might counter this effect and induce the release of virions. This could explain the higher viral load when CD4 is highly downregulated in infected cells [48]. On the other hand, we observed that frequencies of HLA-ABC^low^ remained stable in both p24+ and p24-cells when comparing infection stages. However, the frequencies of HLA-ABC^low^ in p24+ cells inversely correlated with the CD4 count, highlighting a lower HLA-ABC downregulation when CD4 is low, in other words, during AIDS. Similarly, class I major histocompatibility complex (MHC) down-regulation has been previously shown to decrease in the terminal stage progressively [46] and to correlate with the rate of CD4 decline [47].

Furthermore, we showed that p24+ cells significantly displayed a memory (CD45RA-) and a proliferative (Ki67+) phenotype. It has also been demonstrated that both productively and latently infected cells were frequently memory cells [1, 2, 49] and activated cells [6, 21] in peripheral blood and lymph nodes. Here, we showed that their enrichments were detectable but did not vary throughout the infection. However, the frequencies of Ki67+ in total CD4+ T cells were positively correlated with plasma viral load and negatively correlated with CD4 count. This suggests a higher proliferative state in advanced stages of infection. Additionally, the same correlations have been observed for Ki67+ in CD4+ T cells [50], while immune activation (HLA-DR+, CD38+) in CD4+ T cells has been detected as highest in AIDS individuals [34].

Plus, we also identified p24+ as exhausted (PD-1+) cells, which has also been done for productively and latently infected cells in the literature (PD-1+, TIGIT+, LAG-3+, Tim-3+) [4, 6]. More specifically, PD-1 expression in p24+ cells was significantly higher during advanced stages, while that of TIGIT was inversely correlated with the CD4 count in both p24+ and total CD4+ T cells. Altogether, this shows that productively infected cells become more exhausted as infection progresses. Previous studies have indeed shown that PD-1 or TIGIT expression correlates with the CD4 count in total CD4+ T cells [31–33]. This increased exhausted state could result from the repeated stimulations of these cells by the time of the latest HIV stages.

Moreover, we showed that p24+ cells displayed significantly lower frequencies of infected pTfh during AIDS compared to non-AIDS stages. Their enrichment presented the same significant variations. In addition, the frequencies of pTfh in p24+ cells were also inversely correlated with the CD4 count, supporting the idea that productively infected cells are less likely to be pTfh cells during AIDS. Although data about HIV-infected cells and pTfh/Tfh during AIDS are scarce, productively and latently infected cells have been described as being enriched in pTfh cells, with frequencies rising from acute to chronic infection in both blood and lymph nodes [6, 21]. The pTfh cells are thought to be reflective of lymphoid Tfh cells [27], which have been identified as a major reservoir in lymph nodes during chronic infection and under ART [3, 21, 29]. The observed low frequencies of pTfh during AIDS could thus indicate a deletion of Tfh cells, probably due to the progressive lymph node remodeling in advanced stages of infection. Consequently, HIV production might be dealt with by other cells than Tfh in the terminal stage, such as blood cells. A future research perspective would be to assess the contribution of Tfh cells to the pool of productively infected cells in lymph nodes during AIDS.

Lastly, the expression of HIV coreceptors CCR5 and CXCR4 remained stable throughout infection. CXCR4 expression has indeed been shown to remain stable between the stages. Conversely, according to other reports, p24+ cells were not enriched in CCR5, and CCR5 expression remained stable throughout infection [6, 21, 37]. This could be due to some individuals harboring X4 tropic viral strains and the limited number of individuals analyzed. We found no differences in coreceptors expression on infected cells according to HIV tropism on blood T cells [39]. However, our results suggest that preferential CXCR4+ CD4+ T-cell targets were more frequent in participants harboring R5X4/X4-tropic variants when compared to participants harboring R5-tropic variants. Concerning the preferential expression of TIGIT in p24+ R5X4/X4-infected cells, we showed that TIGIT expression is inversely correlated with the CD4+ T-cell count, and all R5X4/X4 infected participants displayed low CD4 counts. We thus believe that TIGIT expression is rather associated with a low CD4 count and not with viral tropism. Overall, we can thus hypothesize that the tropism switch might not be due to a difference in HIV coreceptors expression but is rather due to a difference in the targeted cell populations.

One of the strengths of our study is that phenotypic features of productively infected cells were shared among all participants although they were infected with different HIV-1 subtypes. Of note, the two participants harboring CRF18 strains tended to have higher levels of expression of TIGIT in both p24+ and p24-cells and lower expression of CD4 in total CD4+ T cells. This might be because both of them are in the “AIDS” group and displayed the lowest CD4 counts of all participants.

To more specifically assess the phenotype of these productively infected cells, we studied the markers' co-expression using a UMAP analysis on p24+ and p24-cells. The estimated phenotypes might be incomplete since the expression was analyzed based on a limited number of markers. However, we showed that productively infected cells were enriched throughout the infection in highly differentiated and exhausted cells (memory, memory exhausted, and T_TD_-like) and less likely in naïve cells in peripheral blood. The comparison of each population cell frequencies between HIV stages revealed that p24+ cells remained memory cells all along the infection and tended to be more specifically enriched in memory exhausted cells during advanced stages of infection. Since plasma viral load remains high in AIDS individuals, we can hypothesize that these highly differentiated CD4+ T cells are responsible for HIV production in peripheral blood in the terminal stage. As stated in previous studies, it has indeed been shown that infected memory CD4+ T cells constitute the main reservoir in peripheral blood [1, 49]. In contrast, we observed a significantly low proportion of p24+ cells enriched in naïve cells compared to p24-cells. In accordance with the literature, naïve CD4+ T cells are indeed less preferentially infected by HIV in comparison with memory CD4+ T cells [1, 51]. Although non-significant, a trend of lower naïve cell frequencies was observed as the infection progressed. Altogether, we observed phenotypic changes in productively infected cells throughout infection as disease progressed. We hypothesize these changes might be linked with phenotypic changes in the total pool of CD4+ T cells and to intra-host viral evolution, rather than variation in integration sites landscapes, as these were recently shown not to vary during infection and after ART [52].

To compare productively infected and translation-competent latently infected cells, we added PBMCs from ART-treated individuals to our analysis. The frequency of reservoir cells was expectedly lower than that of productively infected cells [6]. Moreover, latently infected cells were all memory cells (CD45RA-) and were more frequently HLA-ABC+. The absence of downregulation of HLAABC might be due to a different expression level or kinetic activity of nef protein. Interestingly, reservoir cells tended to be less exhausted (PD-1+) than productively infected and should be further explored. Targeting PD-1 has been part of the design of some curative strategies and has shown some benefits in reducing the SIV reservoir size in macaque models [53]. Targeting multiple proteins preferentially expressed by infected cells (as shown here and by others) at the time of viremia and maybe concurrent to ART initiation could strengthen these cure interventions.

Overall, further studies need to be conducted on productively infected cells detected during AIDS to precisely determine their phenotype and the targeted cell populations responsible for HIV pro-duction in peripheral blood and other compartments.

## SUPPLEMENTARY DATA

Supplementary materials are available at the *Pathogens and Immunity* website. Supplementary data may be provided by the authors to benefit the reader. Supplementary data are not copyedited and are the sole responsibility of the authors. Questions or comments related to supplementary materials should be addressed to the corresponding author.
