# Supplementary material for: HIV Productively Infects Highly Differentiated and Exhausted CD4+ T Cells During AIDS
Source: Pathog Immun. 2024 Feb 22;8(2):92–114. doi: 10.20411/pai.v8i2.638 (PMC10901154; doi:10.20411/pai.v8i2.638)
Supplement: Supplementary Data [file pai-8-092-s01.pdf]

PATHOGENS AND IMMUNITY
IN ASSOCIATION WITH CASE WESTERN RESERVE UNIVERSITY AND THE RICHARD J. FASENMYER FOUNDATION

Supplemental Figure 1

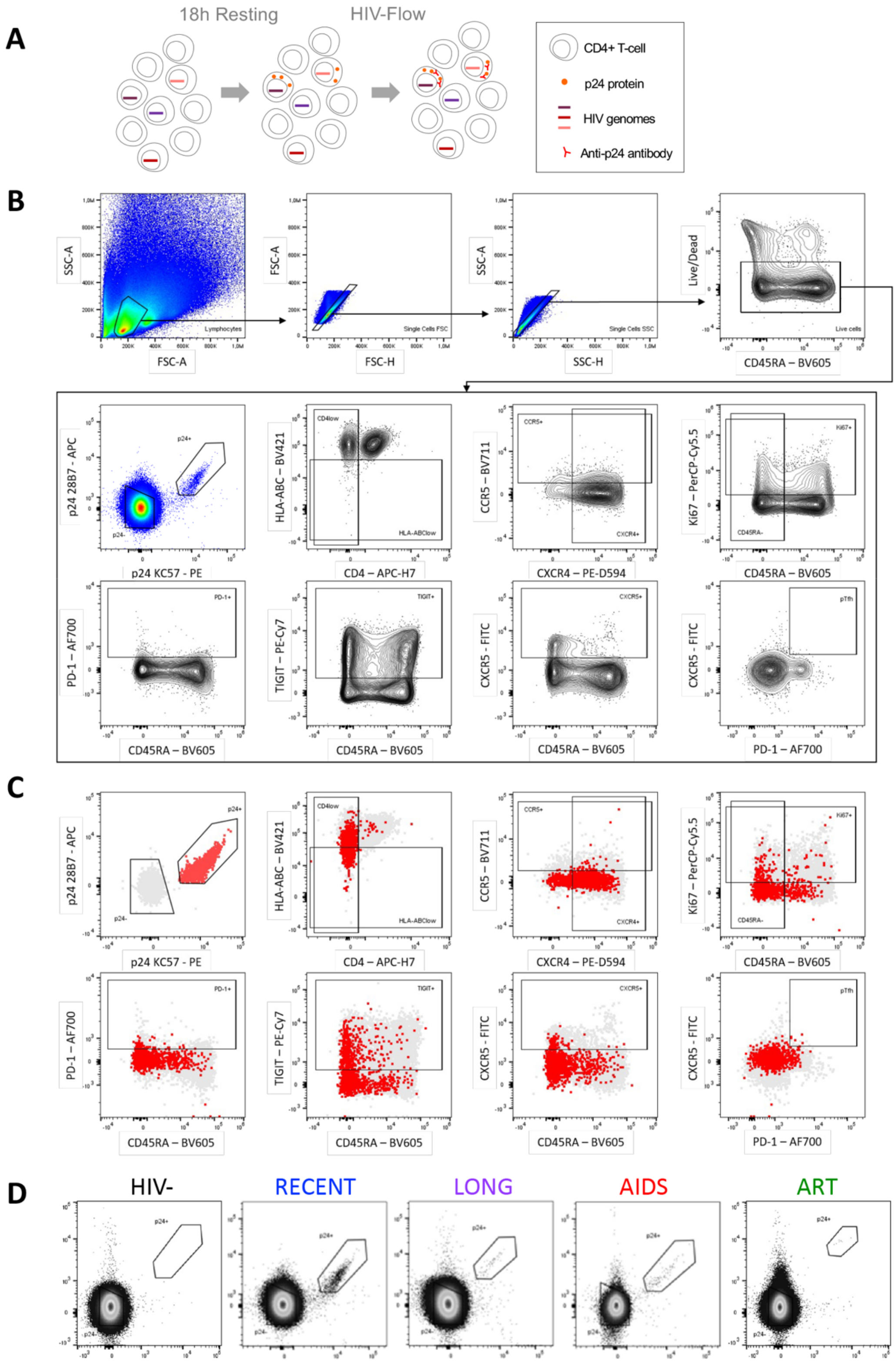

Faua C, Ursenbach A, Fuchs A, Caspar S, Jegou F, Ruch Y, Hoellinger B, Laugel E, Velay A, Rey D, Fafi-Kremer S, Gantner P. HIV Productively Infects Highly Differentiated and Exhausted CD4+ T Cells During AIDS. *Pathogens and Immunity*. 2024;8(2):92-114. doi: 10.20411/pai.v8i2.638

PATHOGENS AND IMMUNITY
IN ASSOCIATION WITH CASE WESTERN RESERVE UNIVERSITY AND THE RICHARD J. FASENMYER FOUNDATION

Supplementary Figure 1. Gating strategy for single cell sorting. A. Isolated CD4+ T cells were rested for 18 h and their p24 expression was analyzed by HIV-Flow. B. Gating strategy used for p24+ cell analysis with recording of CD4, HLA-ABC, CD45RA, CCR5, CXCR4, Ki67, PD-1, TIGIT and CXCR5 expression. C. Representative dot plots showing expression of markers described in B. in p24+ cells (in red), overlaid onto p24- cells (in grey). D. Representative dot plots showing p24+ gating for one participant of each group (HIV-, recent infection, long-term infection, AIDS and ART-treated participants).

PATHOGENS AND IMMUNITY
IN ASSOCIATION WITH CASE WESTERN RESERVE UNIVERSITY AND THE RICHARD J. FASENMYER FOUNDATION

Supplemental Figure 2

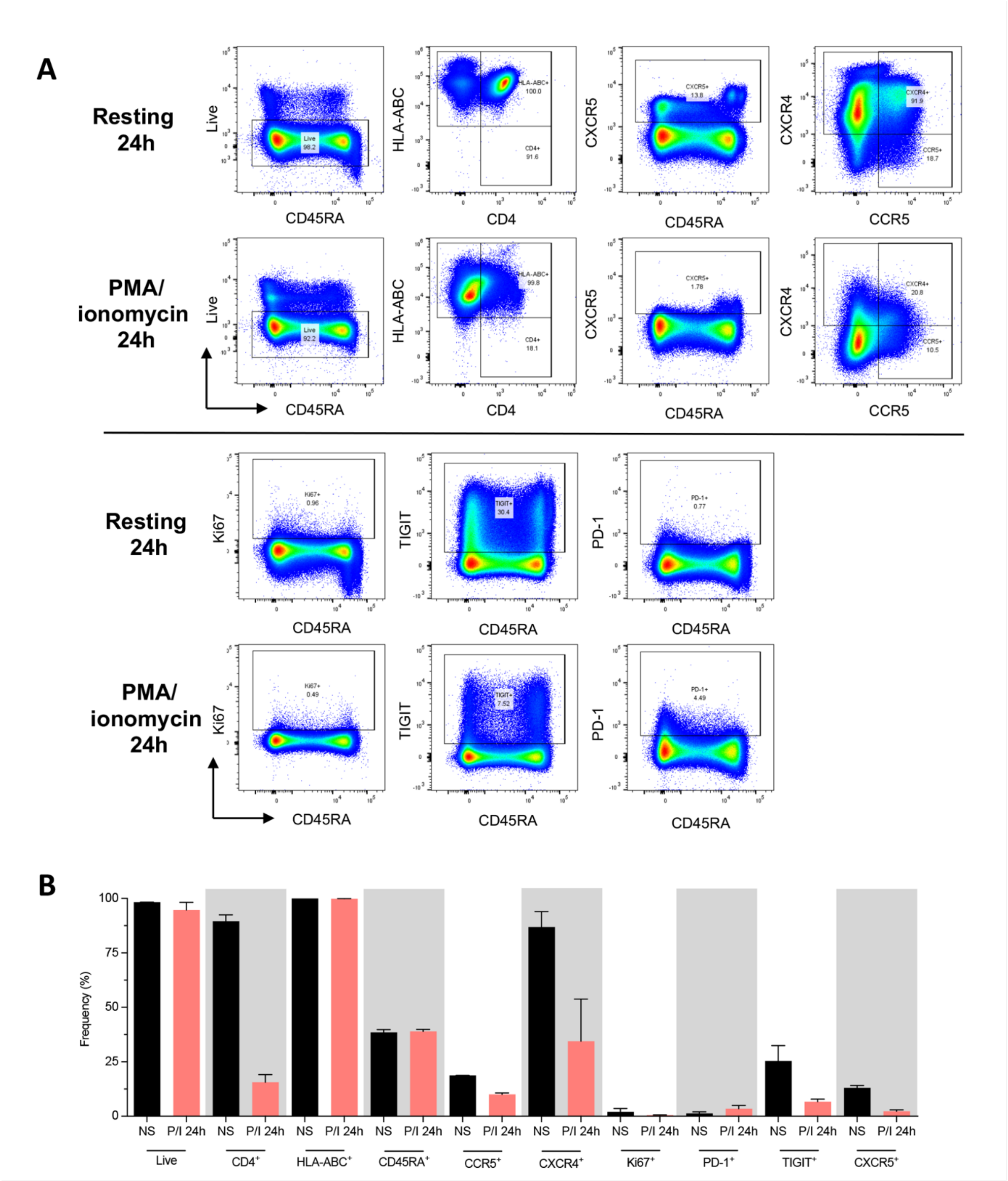

Supplementary Figure 2. Phenotype changes upon stimulation with PMA/ionomycin. A. Representative dot plot from one HIV- individual for each marker of interest in the absence or in presence of stimulation with BFA + PMA/ionomycin. B. Differential phenotypic expression for each marker. CD4, CCR5, CXCR4, TIGIT and CXCR5 were downregulated.

Faua C, Ursenbach A, Fuchs A, Caspar S, Jegou F, Ruch Y, Hoellinger B, Laugel E, Velay A, Rey D, Fafi-Kremer S, Gantner P. HIV Productively Infects Highly Differentiated and Exhausted CD4+ T Cells During AIDS. *Pathogens and Immunity*. 2024;8(2):92-114. doi: 10.20411/pai.v8i2.638

PATHOGENS AND IMMUNITY
IN ASSOCIATION WITH CASE WESTERN RESERVE UNIVERSITY AND THE RICHARD J. FASENMYER FOUNDATION

Supplemental Figure 3

Faua C, Ursenbach A, Fuchs A, Caspar S, Jegou F, Ruch Y, Hoellinger B, Laugel E, Velay A, Rey D, Fafi-Kremer S, Gantner P. HIV Productively Infects Highly Differentiated and Exhausted CD4+ T Cells During AIDS. *Pathogens and Immunity*. 2024;8(2):92-114. doi: 10.20411/pai.v8i2.638

PATHOGENS AND IMMUNITY
In Association with Case Western Reserve University and The Richard J. Fasenmyer Foundation

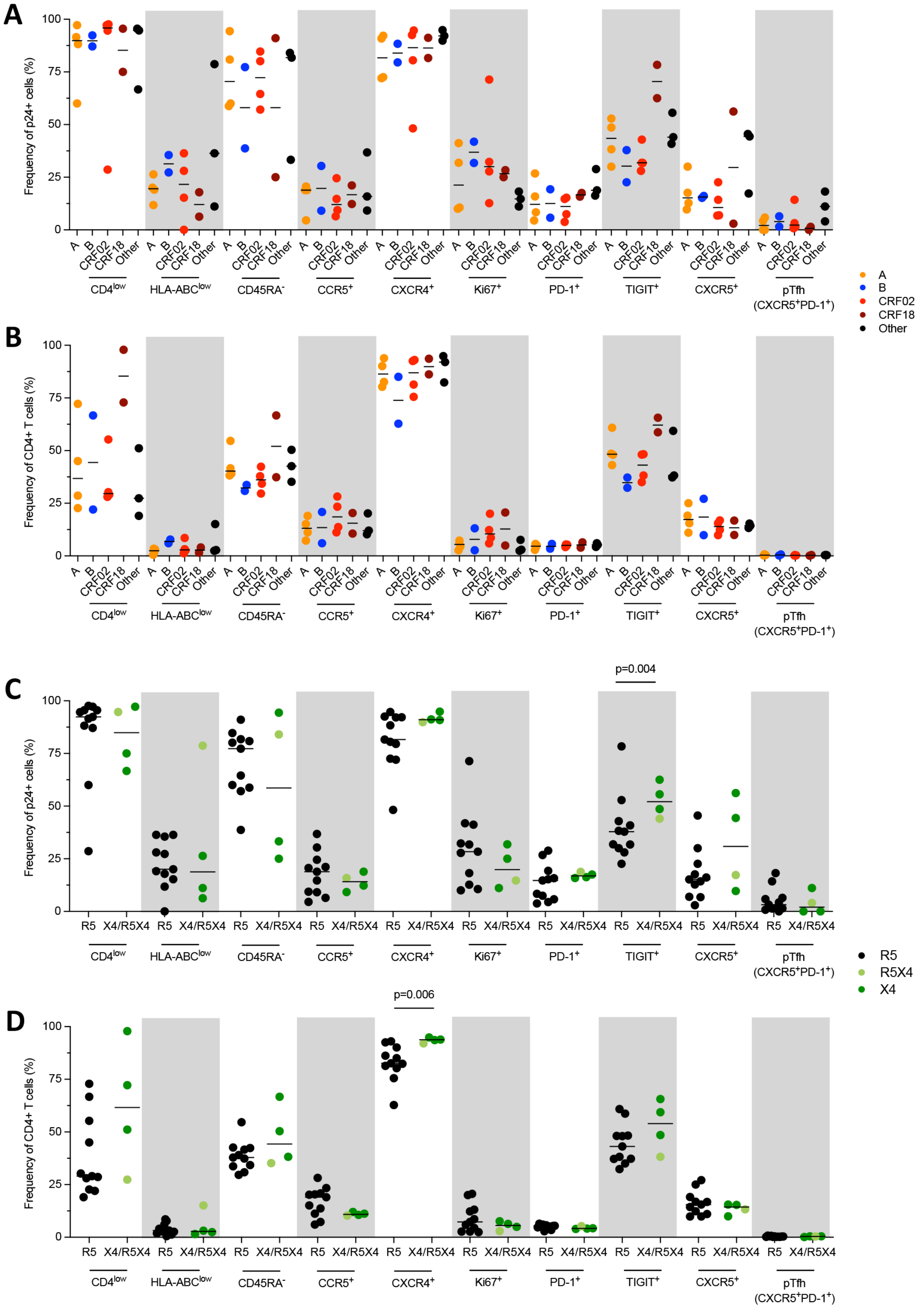

Faua C, Ursenbach A, Fuchs A, Caspar S, Jegou F, Ruch Y, Hoellinger B, Laugel E, Velay A, Rey D, Fafi-Kremer S, Gantner P. HIV Productively Infects Highly Differentiated and Exhausted CD4+ T Cells During AIDS. *Pathogens and Immunity*. 2024;8(2):92-114. doi: 10.20411/pai.v8i2.638

PATHOGENS AND IMMUNITY
IN ASSOCIATION WITH CASE WESTERN RESERVE UNIVERSITY AND THE RICHARD J. FASENMYER FOUNDATION

Supplementary Figure 3. Impact of HIV-1 subtype diversity and tropism on the phenotype of p24+ cells and CD4+ T cells. A-B. Participants were grouped according to their HIV-1 subtype (A, n=4; B, n=2; CRF02, n=4; CRF18, n=2, other, n=3) and the phenotype was displayed for p24+ cells (A.) and CD4+ T cells (B.). No statistical testing was possible due to limited numbers of participants per group. C-D. Participants were grouped according to the tropism of their HIV- 1 strains (R5, n=11; R5/X4, n=1; X4, n=3) and the phenotype was displayed for p24+ cells (C.) and CD4+ T cells (D.). Significant changes are highlighted with the p-value (Mann-Whitney).

PATHOGENS AND IMMUNITY
IN ASSOCIATION WITH CASE WESTERN RESERVE UNIVERSITY AND THE RICHARD J. FASENMYER FOUNDATION

Supplemental Figure 4

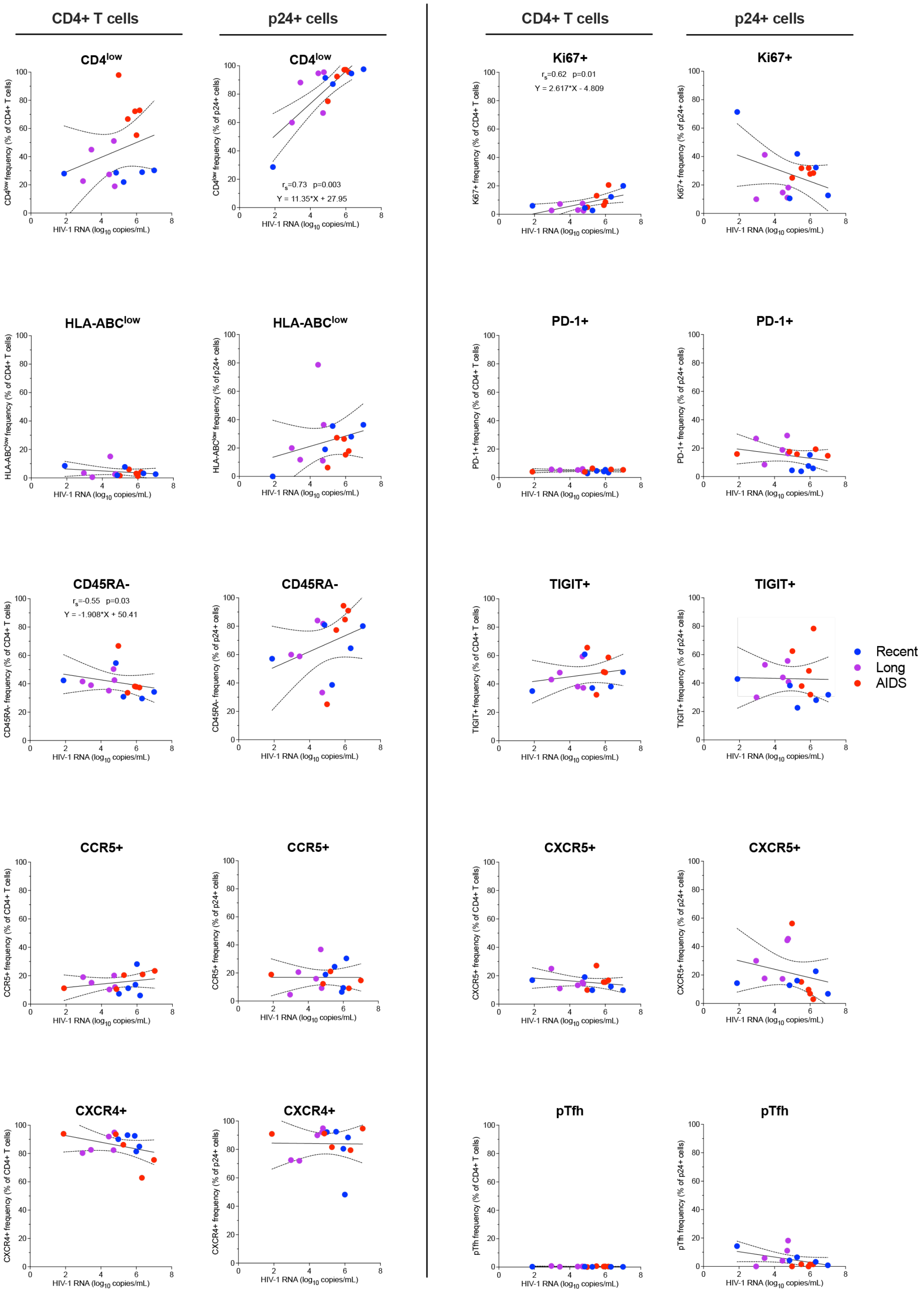

Faua C, Ursenbach A, Fuchs A, Caspar S, Jegou F, Ruch Y, Hoellinger B, Laugel E, Velay A, Rey D, Fafi-Kremer S, Gantner P. HIV Productively Infects Highly Differentiated and Exhausted CD4+ T Cells During AIDS. *Pathogens and Immunity*. 2024;8(2):92-114. doi: 10.20411/pai.v8i2.638

Pathogens and Immunity
In Association with Case Western Reserve University and The Richard J. Fasenmyer Foundation

Supplementary Figure 4. Correlation between markers expression and plasma viral load for viremic participants. Correlations between plasma viremia and the frequency of total CD4+ T cells (left) and p24+ cells (right) expressing or not one the following markers: CD4low, HLA-ABClow, CD45RA-, CCR5+, CXCR4+, Ki67+, PD-1+, TIGIT+, CXCR5+, pTfh cells (CXCR5+PD- 1+). The regression line was plotted with 95%CI. Significant associations are highlighted with the regression equation and correlation values (Spearman).

PATHOGENS AND IMMUNITY
IN ASSOCIATION WITH CASE WESTERN RESERVE UNIVERSITY AND THE RICHARD J. FASENMYER FOUNDATION

Supplemental Figure 5

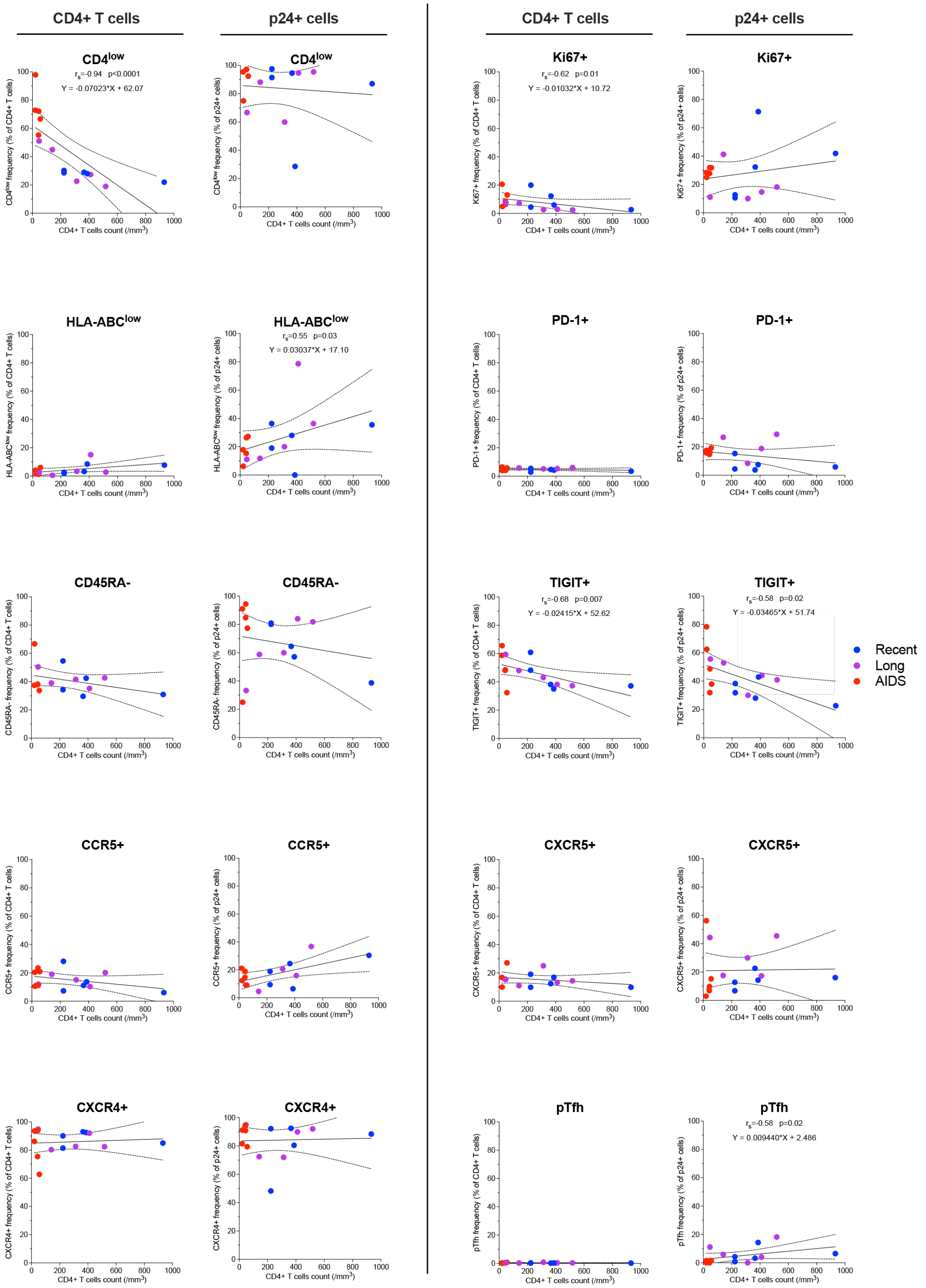

Faua C, Ursenbach A, Fuchs A, Caspar S, Jegou F, Ruch Y, Hoellinger B, Laugel E, Velay A, Rey D, Fafi-Kremer S, Gantner P. HIV Productively Infects Highly Differentiated and Exhausted CD4+ T Cells During AIDS. *Pathogens and Immunity*. 2024;8(2):92-114. doi: 10.20411/pai.v8i2.638

PATHOGENS AND IMMUNITY
IN ASSOCIATION WITH CASE WESTERN RESERVE UNIVERSITY AND THE RICHARD J. FASENMYER FOUNDATION

Supplementary Figure 5. Correlation between markers expression and CD4+ T cells count for viremic participants. Correlations between CD4+ T cells count and the frequency of total CD4+ T cells (left) and p24+ cells (right) expressing or not one the following markers: CD4low, HLA-ABClow, CD45RA-, CCR5+, CXCR4+, Ki67+, PD-1+, TIGIT+, CXCR5+, pTfh cells (CXCR5+PD-1+). The regression line was plotted with 95%CI. Significant associations are highlighted with the regression equation and correlation values (Spearman).

PATHOGENS AND IMMUNITY
IN ASSOCIATION WITH CASE WESTERN RESERVE UNIVERSITY AND THE RICHARD J. FASENMYER FOUNDATION

Supplemental Figure 6

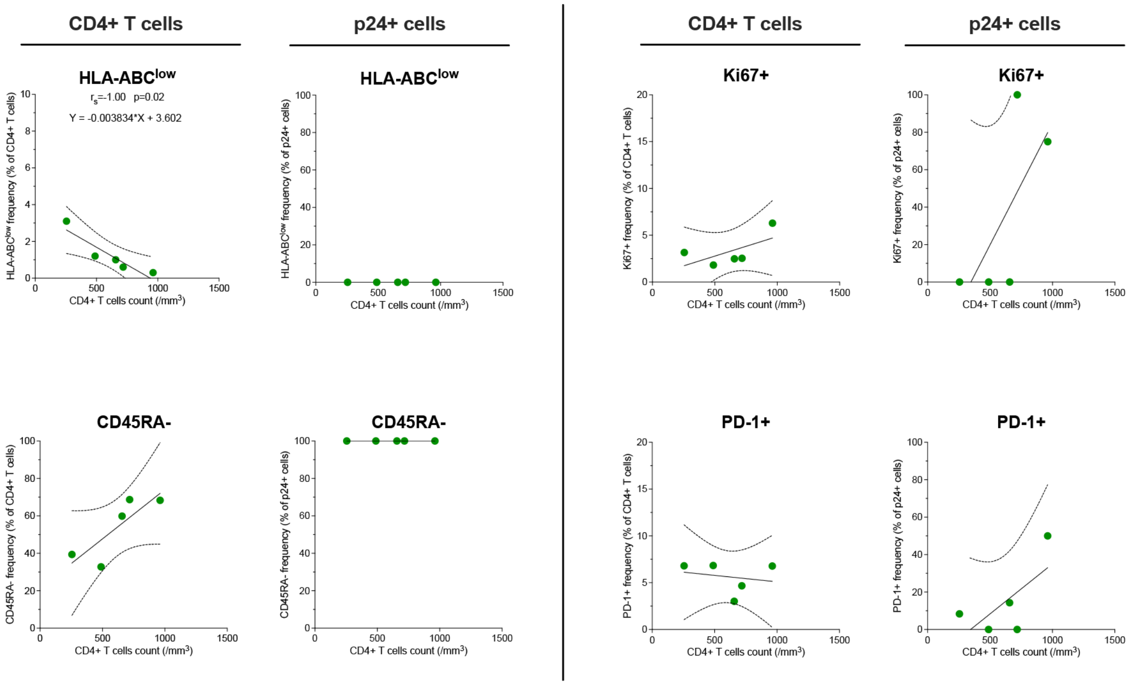

Supplementary Figure 6. Correlation between markers expression and CD4+ T cells count for ART-treated participants. Correlations between CD4+ T cells count and the frequency of total CD4+ T cells (left) and p24+ cells (right) expressing or not one the following markers: HLA-ABClow, CD45RA-, Ki67+, PD-1+. The regression line was plotted with 95%CI. Significant associations are highlighted with the regression equation and correlation values (Spearman).

Faua C, Ursenbach A, Fuchs A, Caspar S, Jegou F, Ruch Y, Hoellinger B, Laugel E, Velay A, Rey D, Fafi-Kremer S, Gantner P. HIV Productively Infects Highly Differentiated and Exhausted CD4+ T Cells During AIDS. *Pathogens and Immunity*. 2024;8(2):92-114. doi: 10.20411/pai.v8i2.638
